# Artificial Intelligence–Based Approaches for Brain Tumor Segmentation in MRI: A Review

**DOI:** 10.1002/nbm.70141

**Published:** 2025-09-17

**Authors:** Khadija Bibi, Mehmood Nawaz, Sheheryar Khan, Muhammad Daud, Anum Masood, Muhammad Ashraf Abdelgawad, Syed Muhammad Tariq Abbasi, Rizwan, Ahsan Khan, Wu Yuan

**Affiliations:** ^1^ Department of Biomedical Engineering The Chinese University of Hong Kong Sha Tin Hong Kong SAR China; ^2^ Department of Applied Data Science Hong Kong Shue Yan University North Point Hong Kong SAR China; ^3^ Division of Science, Engineering, and Health Studies (SEHS), School of Professional Education Executive Development The Hong Kong Polytechnic University Hung Hom Hong Kong SAR China; ^4^ Department of Agriculture Bahauddin Zakariya University Multan Pakistan; ^5^ Harvard Medical School Harvard University Boston Massachusetts USA; ^6^ Department of Electrical Engineering City University of Hong Kong Kowloon Tong Hong Kong SAR China; ^7^ Center for Regenerative Medicine and Health Chinese Academy of Sciences Pak Shek Kok Hong Kong; ^8^ Academy of Wellness and Human Development, Faculty of Arts and Social Sciences Hong Kong Baptist University Kowloon Tong Hong Kong SAR China

**Keywords:** brain tumor segmentation, computed tomography, convolution neural networks, deep learning, foundation models, machine learning, magnetic resonance imaging, transformers

## Abstract

Manually segmenting brain tumors in magnetic resonance imaging (MRI) is a time‐consuming task that requires years of professional experience and clinical expertise. To address this challenge, researchers have proposed artificial intelligence–based strategies that enable quick and automatic segmentation of brain tumors. These AI techniques are crucial for the early identification of brain tumors, leading to earlier diagnoses and significant therapeutic benefits. convolutional neural networks (CNN), vision transformers (ViT), and other automated approaches that leverage machine learning and deep learning techniques have demonstrated effectiveness in diagnosing tumor type, size, and location. Consequently, brain tumor segmentation has emerged as a prominent issue in medical image analysis. This study aims to provide a concise review of MRI techniques and examine popular approaches for segmenting brain tumors. It highlights notable advancements in this field over the past several years. To ensure comprehensive coverage of technical topics, including network architecture design, segmentation in unbalanced settings, and multi‐modality processes, over 200 scholarly publications have been meticulously selected for discussion. Based on this literature review, CNN‐based methods and hybrid approaches have shown exceptional results in segmenting brain tumors from MRI images. Additionally, our study outlines the challenges and potential avenues for future research in brain tumor segmentation techniques.

## Graphical abstract

Manually segmenting brain tumors in magnetic resonance imaging is a time‐consuming task that requires years of professional experience and clinical expertise. We proposed a study, which contains a comprehensive review of the brain tumor segmentation techniques. It selects the effective approaches to better understand the AI applications for brain tumor detection. It gives potential directions such as foundation models and real‐time tumor segmentation in 3D. Our study also outlines the potential avenues of brain tumor segmentation techniques for future research.

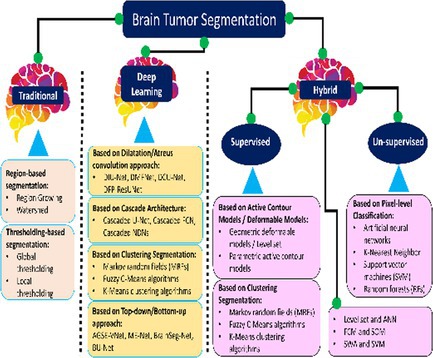

**Abbreviations**
AI: artificial intelligence
CNN: convolutional neural networks
CSF: cerebrospinal fluid
CT: computed tomography
DMF: dilated multi‐fiber
DWA: dynamic window approach
ENet: element net
FCM: fuzzy c‐mean
FCN: fully convolutional network
FN: false negative
FP: false positive and
GA: genetic algorithm
GM: gray matter
HD: Hausdorff distance
HGG: high‐grade gliomas
IEEE: Institute of Electrical and Electronics Engineers
KNN: K‐nearest neighbor
LGG: low‐grade gliomas
MF: multi‐fiber
MRF: Markov random field
MRI: magnetic resonance imaging
MSE: mean square error
OCT: optical coherence tomography
PET: positron emission tomography
ReLU: rectified linear unit
RF: random forest
ROI: region of interest
SLIC: simple linear iterative clustering
SVM: support vector machine
TNet: tumor network
TP: true positive
ViT: vision transformers
WM: white matter
WNet: window net

## 1 Introduction

**FIGURE 1:**
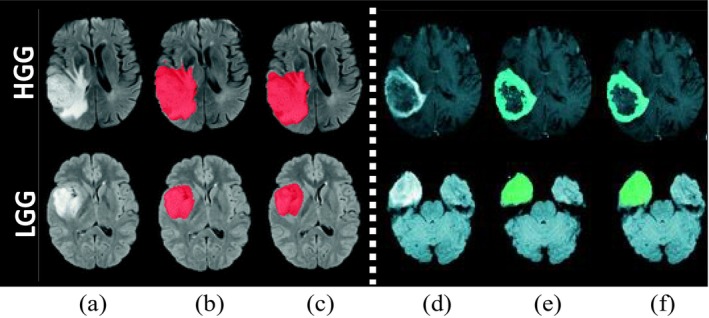
The segmentation results for HGG and LGG cases compared with manually generated ground truth; (a and d) columns are original MRI images, (b and e) columns are the ground truth of MRI images, and (c and f) columns are the segmented results of tumor (red) and enhanced tumor area (cyan) using deep learning technique.

Medical image segmentation has been a popular topic for the past two decades [1, 2, 3]. Various imaging approaches, including X‐ray, ultrasound, magnetic resonance imaging (MRI), positron emission tomography (PET), and computed tomography (CT), have been utilized for segmenting the tumors, disease detection, diagnosis, and treatment. Among these imaging methods, MRI has particularly proven to be beneficial in clinical practice for evaluating gliomas due to its ability to provide additional information through various MRI sequences. Figure 1 demonstrates the difference between high‐grade (HGG) and low‐grade (LGG) gliomas. It shows that HGG are more aggressive and invasive compared to LGG.

Expert radiologists are typically associated with the applications of MRI images. However, both academics and medical professionals have begun to rely on computer‐assisted therapies due to the distinction in pathology and the potential limitations of human expertise [4]. Recently, deep learning‐based medical imaging techniques have grown rapidly to analyze any medical modality [5, 6, 7, 8, 9]. Accurate extraction of important features and details from medical images (2D and 3D images) is essential for segmentation.

Medical image segmentation aims to divide an image into distinct and comprehensive parts to explore the more features hidden inside the image. These parts should be physically connected and have uniform pixels, based on a predetermined criterion typically related to intensity levels. Homogeneity can also be assessed in these sections using other factors such as surface normal, texture, color, range, and surface curvature [10]. However, this definition presents a significant constraint when identifying abnormal tissue and detecting malignancies in brain MRI images [11]. The anatomical structures that are segmented vary in location and structure from one patient to another, and they frequently have complicated and flexible shapes. The brain has two basic types of tissues: One is normal tissue, and the second is abnormal tissue, which is called tumor (active tumor, edema, and necrosis tumor). The deep learning techniques first identify the normal tissues to classify the abnormal tissues [12].

### 1.1 AI Applications in MRI Clinical Research

AI applications have greatly advanced in MRI clinical research that significantly improve data capturing, reconstructing, and analyzing in the MRI fields [13]. One major benefit of AI is its ability to speed up MRI scans by reducing the number of measurements needed, leading to faster imaging while maintaining high‐quality results. AI methods also enhance image reconstruction, producing clearer images with fewer distortions. Furthermore, AI‐powered noise reduction and super‐resolution techniques improve MRI image quality, making it easier for radiologists to interpret and diagnose conditions.

AI‐driven automated segmentation accurately identifies anatomical structures and pathological features, such as tumors or lesions, enabling precise and consistent quantitative analysis [14]. This leads to more accurate evaluations of disease progression, treatment responses, and intervention planning. Machine learning algorithms can detect patterns and radiomics features to identify biomarkers, predict clinical outcomes, and personalize treatment plans. AI also improves workflow by prioritizing urgent cases, automating routine tasks, and assisting doctors in decision‐making. It enhances the integration of different imaging types and long‐term studies, allowing for detailed analysis and tracking over time. These capabilities not only boost diagnostic accuracy and efficiency but also aid in clinical trial research and development, leading to new MRI techniques and improved patient outcomes.

**FIGURE 2:**
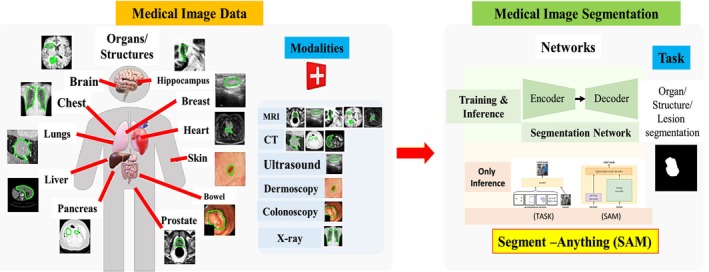
The applications of the foundation model for multiple modalities. The segment anything model (SAM) network contains an encoder and decoder module. It can segment multiple organs using different modalities [21].

The use of MRI is crucial for numerous neurology applications, such as quantitative analysis [15], perfusion imaging [16, 17], and monitoring disease progression [18, 19], as MRI plays a key role in identifying brain structures and obtaining valuable information on soft tissues. However, the process of image segmentation becomes challenging due to low contrast, inhomogeneous intensity levels, and fluctuations in intensity range between MRI sequences [20]. Despite the existence of various image segmentation techniques (Table 1) categorized by academics, no standardized method can consistently produce accurate results. Since the segmentation goal varies depending on the research and the type of image data used, multiple methodologies are employed, each based on a unique understanding of the nature of the images under examination. Recently, foundation models [21, 22] have been proposed to segment the multi organs from multi modalities, as shown in Figure 2. Segmentation techniques can be categorized in numerous ways depending on the classification method used. Segmentation methods in MRI can further be categorized according to the use of either tumor segmentation or brain region segmentation. The choice of method depends on the medical imaging problem and the complexity of the structures to be identified in the MRI data.

**TABLE 1:** Summary of the last decade (2013–2025): significant research on brain tumor segmentation using different techniques, including machine learning, deep learning, and hybrid techniques.

Article	Publisher	Year	Applications
Deep learning models and traditional automated techniques for brain tumor segmentation in MRI: a review	*Artificial Intelligence Review*	2025	This survey focuses on studies done in brain MRI.
Signaling pathways in brain tumors and therapeutic interventions	*Signal Transduction and Targeted Therapy*	2024	Brain tumors contribute to distinct mortality and morbidity at all ages
Deep learning based brain tumor segmentation: a survey	*Complex & Intelligent Systems*	2022	Brain tumor segmentation is one of the most challenging problems
Automated brain tumor segmentation using multimodal brain scans: a survey based on models submitted to the BraTS 2012–2018 challenges	*IEEE Reviews in Biomedical Engineering*	2023	A review of challenge submissions of BraTS during 2012–2018.
A survey on brain tumor detection using image processing techniques	*7th International Conference on Cloud computing. Data science & Engineering‐ confluence*	2017	A review of general brain tumor segmentation methods.
Survey of brain tumor segmentation techniques on magnetic resonance imaging	*Nano Biomedicine and Engineering*	2019	A general summary of classic brain tumor segmentation methods.
State of the art survey on MRI brain tumor segmentation	*Magnetic Resonance Imaging*	2013	Review on convolutional neural networks used for brain MRI image analysis.
A survey of MRI‐based brain tumor segmentation methods	*Tsinghua Science and Technology*	2014	Review on MRI based brain tumor segmentation methods.
Data augmentation for brain‐tumor segmentation: a review	*Frontiers in Computational Neuroscience*	2019	Analyzed the technical details and impacts of different kinds of data augmentation methods with the application to brain tumor segmentation.
A survey on deep learning in medical image analysis	*Medical Image Analysis*	2017	A comprehensive review on deep learning based medical image analysis.
Deep convolutional neural networks for brain image analysis on magnetic resonance imaging: a review	*Artificial Intelligence in Medicine*	2018	A review on use of deep convolutional neural networks for brain image analysis.
Deep learning for brain MRI segmentation: state of the art and future directions	*Journal of Digital Imaging*	2017	A survey on deep learning for brain MRI segmentation.
A guide to deep learning in healthcare	*Nature Medicine*	2019	A survey on deep learning for health‐care.
Deep learning for generic object detection: A survey	*International Journal of Computer Vision*	2020	A comprehensive review on deep learning based object detection.
Deep learning	*Nature*	2015	An introduction review on deep learning and its application.
Recent advances in convolutional neural networks	*Pattern Recognition*	2018	A survey on convolutional neural networks and its application on computer vision, language processing and speech.

FDA has approved many AI medical tools for MRI brain scans, which enhance the accuracy and speed of brain tumor segmentation in MRI images [23]. There are some FDA‐approved AI devices including (1) Pixyl Neuro, which analyzes MRI brain scans to detect and monitor disease activity in conditions like multiple sclerosis and other neuroinflammatory and neurodegenerative disorders; (2) Rapid ASPECTS, which is developed by iSchemaView, assists in evaluating brain CT and MRI scans to support the diagnosis of stroke and other neurovascular conditions; (3) MAGNETOM Sola and MAGNETOM Altea, which are AI‐enhanced MRI systems that are developed by Siemens Medical Solutions to improve image quality and workflow efficiency in radiological practices; (4) LungQ, which is made by Thirona BV; it helps in analyzing lung MRI scans, lung disease detection and monitoring; and (5) Butterfly iQ3, which is an AI‐enabled portable ultrasound system developed by Butterfly Network. It supports various imaging needs, including MRI interpretations.

### 1.2 Challenges of AI Models in MRI Clinical Research

AI models have great potential for use in clinical MRI image studies, but they face several challenges that need to be resolved before they can be widely and effectively used [24]. Here are some of the main challenges.

#### 1.2.1 Data Quality and Quantity

The quality and quantity of data pose major challenges for AI in MRI clinical research. High‐quality, labeled datasets are rare and costly, requiring significant cooperation with medical institutions and radiologists. Furthermore, it is challenging to apply AI models broadly in clinical settings due to variations in MRI procedures, scanner types, and patient demographics that produce noisy data. Inconsistent data labeling also decreases model dependability and performance. Limited data can lead to overfitting, which occurs when models perform well on training data but not on fresh data. To address these issues, it is critical to standardize data gathering, develop high‐quality labeled datasets, and assure variety and consistency for effective AI applications.

#### 1.2.2 Annotation and Labeling

MRI image annotation and labeling are a difficult task in clinical research for AI models. Accurate labeling necessitates the expertise of radiologists, which takes time and money. Additionally, various radiologists may categorize the same images differently, resulting in inaccuracies and hurting the AI's effectiveness. High‐quality, consistent annotations are essential for training effective AI models, but obtaining them is difficult due to the subjective nature of medical image interpretation. To increase AI accuracy and efficacy in healthcare, we need improved annotation techniques and tools that provide accurate and consistent training data.

#### 1.2.3 Model Interpretability and Transparency

AI model interpretability and openness are important considerations in MRI clinical applications. Many AI models, particularly deep learning models, function as “black boxes,” making decision‐making processes difficult to explain. This lack of transparency may impair physicians' trust and desire to utilize these models, as medical practitioners must interpret and validate AI‐generated outcomes. To properly incorporate AI models into healthcare, the results must be intelligible and transparent. Techniques for explaining model predictions can increase confidence and allow clinicians to make educated judgments based on AI‐assisted insights.

#### 1.2.4 Integration With Clinical Workflow

Integrating AI models into clinical MRI operations is a substantial hurdle. These AI solutions must integrate easily into existing processes while not interfering with radiologists and physicians regular activities. They must be user‐friendly, interoperable with existing health IT systems, and capable of delivering real‐time support. Furthermore, sufficient training and support are required to guarantee that healthcare professionals can properly use these AI technologies. When done right, this integration increases efficiency, helps with decision‐making, and ultimately improves patient care. However, attaining this needs meticulous preparation and collaboration among multiple healthcare centers.

#### 1.2.5 Clinical Relevance of Segmentation

Precise segmentation of brain tumors is clinically valuable and supports important tasks, including predicting the prognosis, targeting radiation therapy, and surgical planning. By defining tumor subregions, such as necrotic tissue, peritumoral edema, and tumor core, it is possible to more accurately track tumor growth or response to treatment. Clinical operations may become more consistent and efficient using AI‐based segmentation, as shown by the tools created for the BraTS challenge. In addition, segmentation outputs are being increasingly incorporated into survival prediction models and quantitative radiomic pipelines, underscoring their growing importance in decision support systems that neurosurgeons, radiologists, and oncologists use in the actual treatment of patients.

#### 1.2.6 Regulatory and Ethical Challenges

Artificial intelligence models in MRI clinical research face substantial regulatory and ethical hurdles. Obtaining regulatory clearance requires significant validation to verify their safety and efficacy, which may be a time‐consuming and difficult procedure. Ethically, it is critical to protect patient privacy, secure data, and remove any biases in AI models that may result in unequal healthcare results. Furthermore, AI decision‐making must be transparent in order to maintain confidence. To solve these problems, AI models must closely comply with legal requirements and ethical principles, ensuring that they are fair, unbiased, and preserve patient confidentiality.

### 1.3 Types of Brain Tumor Segmentation in MRI Images

There are three basic types of brain tumor segmentation in MRI images. The details of each type are given below.

#### 1.3.1 Manual Segmentation of MRI Images

**FIGURE 3:**
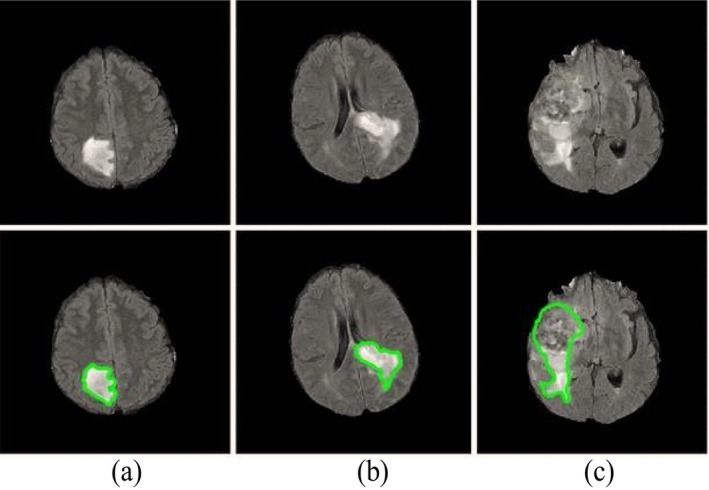
Examples of manual segmentation of brain tumors in MRI images. The first row displays the original images, and the second row shows the manually segmented brain tumor [28].

Manual segmentation of tumors in medical images involves human interaction, either it can be fully manual or semi‐automatic [25, 26]. The level of human interaction required is determined by the amount of manual or semi‐automatic involvement, particularly in MRI image segmentation [27]. The term “manual segmentation” refers to the procedure performed by the radiologist, as shown in Figure 3.

The image is divided and tagged manually, requiring radiologists to enhance their ability to interpret the multi‐modality data provided by MRI scans [29], while also utilizing their anatomical and physiological knowledge gained through education and experience. Both the borders of brain tumors and the identification of anatomical structures must be painstakingly drawn and labeled by hand [25, 30]. However, due to its reliance on human specialists, this manual segmentation approach is more susceptible to errors. The outcomes of manually performed segmentation can differ from one individual to another, as they depend on the operator's knowledge and competency.

Manual segmentation takes a long time to segment the tumor area in the brain MRI image. Researchers are working to make semi‐automatic and full‐automatic brain tumor segmentation techniques to increase efficiency and decrease segmentation time [31]. In semi‐automatic brain tumor segmentation, human intervention is frequently necessary to initiate the process, validate the accuracy of the findings, and manually refine the segmentation for improved efficacy. However, user involvement can lead to variations in segmentation outcomes among individuals. The interactive component is deemed one of the crucial factors in the semi‐automatic brain tumor segmentation approach, as stated by Gordillo et al. [25] and Liu et al. [30]. Nonetheless, completely automatic brain tumor segmentation does not necessitate any user input.

To achieve automated segmentation, researchers used a combination of machine learning techniques and manually labeled data. Fully automatic techniques depend on the prior information of brain MRI images like the position of the tumor, size, and shape. Additionally, a model is required that not only defines the shape, size, and position accurately but also allows for adjustment in response to anticipated changes in characteristics [25].

#### 1.3.2 Supervised Segmentation

**FIGURE 4:**
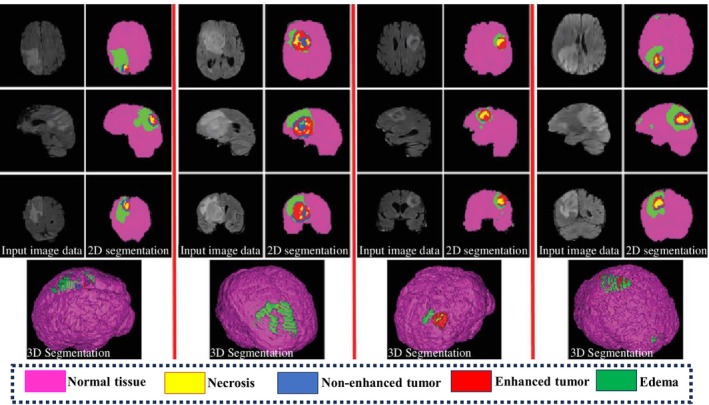
The supervised technique for 2D and 3D brain tumor segmentation using BraTS and Flair‐MRI image datasets. The first, third, fifth, and seventh columns show the input images of coronal, axial, and sagittal planes. The second, fourth, sixth, and eighth columns show the segmentation results of brain tumors (necrosis, non‐enhanced tumor, enhanced tumor, and edema) [37].

Segmentation techniques can be classified as either supervised or unsupervised, depending on the presence of manually labeled training data [32, 33, 34, 35, 36]. In supervised techniques, labeled data are utilized during the training stage to construct a model that links retrieved characteristics with labels or classes. Subsequently, these labels are employed in the testing process to determine the appropriate label for the data [27]. Figure 4 shows an example of a supervised image segmentation technique for 2D and 3D brain tumor segmentation [37].

To ensure optimal results in supervised segmentation, it is imperative to employ the appropriate training data. Using diverse training data may lead to considerable discrepancies during the training process as well as potential differences in the segmentation output [25, 38]. Consequently, manual intervention becomes necessary, which may result in uneven outcomes. Nevertheless, supervised segmentation approaches can eliminate the need for user intervention by providing sufficient labeled data, features, and appropriate settings for the learning algorithm.

#### 1.3.3 Unsupervised Segmentation

Unsupervised segmentation stands apart from supervised segmentation because it does not rely on labeled training data to carry out the segmentation process. Instead, an algorithm is employed to automatically determine the number of classes. To address more intricate circumstances, the image is divided into uniform sections based on image‐dependent variables, such as pixel intensity [34, 39, 40]. Unsupervised segmentation approaches have been proven effective in properly segmenting diverse tumor areas in complex scenarios [41].

This study thoroughly examines the most widely used strategies for tumor segmentation in MRI images. While the reference papers [25, 27, 30] provide comprehensive information on various segmentation techniques, each method possesses limitations and benefits. Consequently, current approaches hold significant potential to make substantial contributions, particularly in the realm of brain tumor segmentation. Furthermore, the purpose of this study is to summarize the state‐of‐the‐art machine learning, deep learning, and hybrid techniques, specifically focusing on the development of CNN blocks for tumor segmentation in brain MRI images. This opens up new research opportunities for future researchers.

We have compiled this study using a wide range of publications and scientific research reports. We gathered information on brain tumor segmentation strategies from reputable sources, including Scopus and Web of Science. Additional resources, such as Science Direct, IEEE Xplore, Springer, Frontiers, and Hindawi, were also utilized. The chosen databases of brain‐related conferences and journal publications were subjected to inclusion and exclusion criteria. Only papers meeting the following requirements, such as no duplicates, full‐text access, and being authored in English, were considered. Table 1 shows the summary of recent survey papers on brain tumor segmentation in MRI images. We selected 180 publications from 200 after scanning the relevant databases and removing the duplicate studies.

##### 1.3.3.1 Contributions

The main summary of our review article includes the following:

- **Summary of significant brain tumor segmentation techniques:** It provides a comprehensive overview of the brain tumor segmentation techniques and analyzes the commonly used methods for segmenting tumors in MRI scans using machine learning, deep learning, and hybrid approaches.
- **Summary of AI applications in clinical research:** This survey highlights the AI models in clinical trials, which help the data analysis, predict outcomes, accelerate drug discovery, and personalize treatments. It explains the improvement of clinical trials, research efficiency, and accuracy.
- **Detailed review and challenges of brain tumor segmentation techniques:** This survey is based on a detailed analysis of numerous scientific papers that focus on the existing techniques for brain tumor segmentation. It discusses the challenges associated with these techniques and proposes various strategies to overcome them.
- **Selection of effective approaches:** The selection of effective approaches becomes easier when researchers utilize both qualitative and quantitative evaluation methods for machine learning and deep learning‐based approaches. This helps in gaining a better understanding of how artificial intelligence can be applied to brain tumor segmentation.
- **Directions for future research:** To guide future research, we have explored novel concepts for potential directions, such as foundation models [22], real‐time tumor segmentation, 3D brain tumor segmentation, and so forth.

The rest of this survey is structured as follows: Section 2 presents a brief overview of the MRI modality for brain tumors, and Section 3 explores different techniques for segmenting brain tumors, including traditional methods, deep learning, and hybrid approaches. Section 4 discusses the performance comparison of brain tumor segmentation techniques. Section 5 explains the future directions of brain tumor studies for researchers. Lastly, Section 6 summarizes the findings of all discussed survey articles about brain tumors in MRI.

## 2 Other Brain Tumor Segmentation Techniques

**FIGURE 5:**
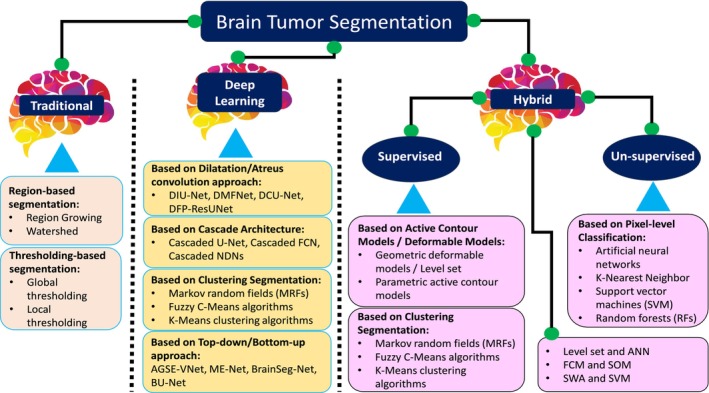
The flow chart of proposed survey work. The automatic brain tumor techniques are divided into three categories: traditional techniques, deep learning, and hybrid techniques. The hybrid techniques are the integration of machine learning and deep learning.

The principal objective of image segmentation is to partition an image into coherent parts according to some given criteria. For brain tumor segmentation, a tumor is separated by distinguishing it from healthy brain tissue that consists of cerebrospinal fluid, including edema, necrosis, and active tumors [25]. During brain tumor segmentation, there are typical approaches used to obtain an objective metric for analyzing the regularity of each tissue. The brain tumor techniques are divided into three main categories: traditional, deep learning, and hybrid approaches, as shown in Figure 5.

### 2.1 Traditional Techniques

Traditional methods for brain tumor segmentation have been extensively used in the field of 2D image segmentation and have shown promising outcomes in boundary delineation and tumor region segmentation. The summary of the most common machine‐learning approaches for brain tumor segmentation is shown in Table 2 [48]. These traditional image processing techniques involve threshold‐based techniques [49], region‐based, watershed [50], SVM, super‐pixel [51], clustering [52], random forest (RF) [53], and Markov random field (MRF) [54].

**TABLE 2:** Overview of traditional brain tumor segmentation methods.

Ref	Year	Algorithm	Segmentation
[42]	2023	SynthSeg+ AI model	MRI brain tumor
[43]	2022	Multi‐resolution handcrafted feature	MRI brain tumor
[25]	2018	Threshold approach	MRI brain tumor
[44]	2018	Support vector machine (SVM)	MRI brain
[45]	2017	Threshold approach	MRI brain tumor
[46]	2017	Super pixel	MRI brain
[30]	2014	SVM, K‐means	CT, MRI brain tumor
[46]	2013	Clustering	MRI brain tumor
[47]	2009	Watershed	MRI brain tumor

#### 2.1.1 Thresholding‐Based Technique

**FIGURE 6:**
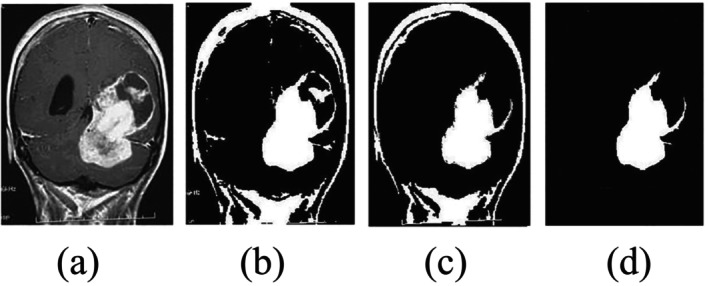
The threshold‐based brain tumor segmentation. (a) The original image, (b) the output results on the threshold (T = 1), (c) the segmentation results on T = 2, and (d) the final output [55].

Conventional threshold‐based segmentation can be a quick, simple, and quite successful method that compares pixel intensity or pixel value, as shown in Figure 6. These can be divided into two approaches: “global thresholding” and “local thresholding” [25, 30, 56].

- **Global thresholding:** It is based on intensity value, allowing an intensity ratio to be found between objects and background in a particular region [57]. Global thresholding is proper when MR images contain objects with almost the same contrast or intensity [44, 45]. The mathematical form of a global thresholding algorithm is expressed in Equation (1).

In Equation (1), $I\left(x,y\right)$ is the input image, $f\left(x,y\right)$ is the thresholded form of the input image, and the calculated threshold value is represented as $f\left(x,y\right)=f\left(T_{0},I\left(x,y\right)\right)$, where $T_{0}$ is the initial threshold value.

(1) $$f\left(x,y\right)=\left\{\begin{array}{ll} 1 & :\;I\left(x,y\right)>T_{0}\\ 0 & :\text{otherwise} \end{array}\right.$$

One of the most common and practical techniques applied in global thresholding is using a universal threshold value for the whole image. This technique is mainly applied in segmenting those images that have objects with homogeneous intensity and indeterminate shapes [58]. The threshold technique, especially in MR images, has one major disadvantage. It is only interested in intensity and not the connectivity of pixels. As a result, when the intensity levels of two or more tissue structures coincide, this method can be inapplicable.

- **Local thresholding:** Local thresholding can be effective for a case in which a histogram fails to find a threshold value for the whole image or when the contribution of the gradient is small compared to the size of the image under consideration [59]. It applies when a single threshold alone cannot give good segmentation results [44, 45]. According to Saman et al. [56], the threshold value p depends on the gray levels of $I\left(x,y\right)$ and other local image features of neighboring pixels, which could be far or nearby. Instead of the whole image $I\left(x,y\right)$, threshold $p_{0}$ is calculated for a pixel through its neighbors within a neighborhood Np. A local thresholding function $f\left(x,y\right)$ is given mathematically by Equation (2).

(2) $$f\left(x,y\right)=\left\{\begin{array}{ll} 1 & :\;I\left(x,y\right)>p_{0}\\ 0 & :\text{otherwise} \end{array}\right.$$

where $p_{0}=f\left(T\in \mathrm{Np}\left(x_{p},y_{p}\right)\right)$.

#### 2.1.2 Morphological Techniques

Region‐based segmentation algorithms analyze pixels in an image based on a similarity criterion and group neighboring pixels with similar properties into disjoint portions [60]. Two region‐based methods include “watershed” and “region growing.”

**FIGURE 7:**
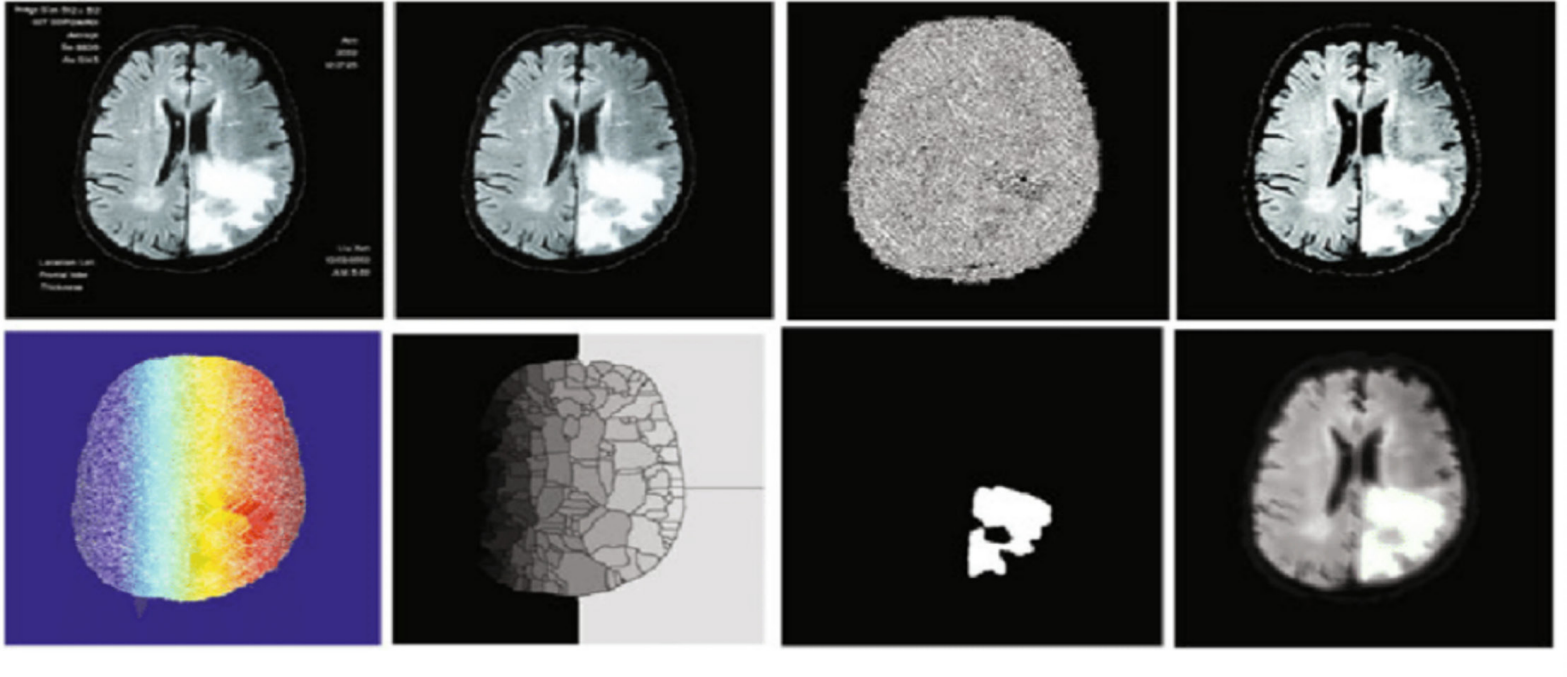
Watershed segmentation results. In the first row (left to right): T1‐MRI images, edge smoothing filter result, bilateral filter‐based de‐noise image, and reduction of impulse noise. In the second row (left to right): gradient image, watershed image, tumor segmentation, and whole image with tumor area.

- **Region growing**: One popular way to partition an image is through the process of selecting pixels or voxels based on their intensity level [61, 62, 63]. This method is initialized with a starting point as a seed that defines the Region of Interest (ROI), and surrounding pixels are measured against the similarity criteria, after which they are appended to the region being grown. The expansion process is repeated until one cannot add any pixels or voxels further to the region.
- **Watershed**: Watershed segmentation is an image morphology‐based region‐based approach used in object segmentation in images, as shown in Figure 7. The underlying concept of a watershed is inspired by the behavior of water in natural landscapes. Similar to how raindrops accumulate in catchment basins in different locations based on water volume, image regions in the watershed approach are associated with catchment basins. Each basin is connected to several valleys, with each valley being exclusively linked to a particular catchment basin. Consequently, each point in the image is part of a separate basin [64].

#### 2.1.3 Superpixel‐Based Segmentation

**FIGURE 8:**
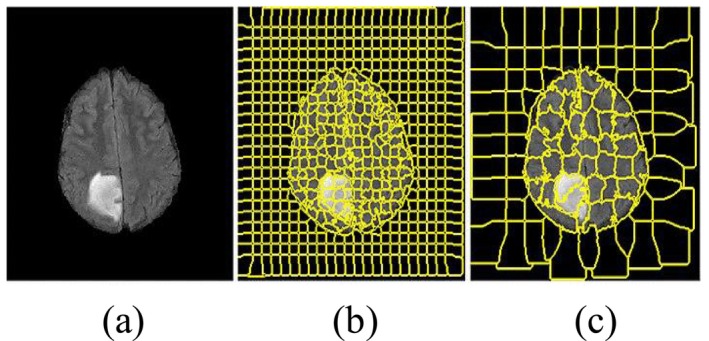
The superpixel‐based segmentation of brain tumor. (a) The input FLAIR MRI image of the grade II tumor, (b) the superpixel segmentation of the MRI image with different thresholds (m = 0.2, S = 10, and grid size 10 × 10), and (c) the superpixel segmentation at S = 20, m = 0.3 and grid size 20 × 20 [28].

The main objective of the superpixel technique is to group those pixels that have the same low‐level attributes into some meaningful visual entities [28, 65]. In this method, simple linear iterative clustering (SLIC) is used for image segmentation to generate superpixels that are smaller patches in an image [66], as represented in Figure 8. This process helps in reducing the large number of pixels to a small number of superpixels to make it faster in computation.

Soltaninejad et al. [28] developed a method to segment brain tumors from MRI images in their research. The method classified each superpixel according to the superpixel algorithm. Within their method, to get a proper classification, image features obtained from each superpixel across the complete region of the brain in FLAIR MRI included intensity‐based attributes, curvatures, Gabor textons, and fractal analyses, among others. More recently, Imtiaz et al. [51] showed that they extracted attributes at the superpixel level from 3D brain MR images for tumor segmentation. Each image is divided into superpixels, irregular patches with more or less uniform intensity and spatial proximity. The core idea of the approach is to minimize dispersed pixels and accurately consider the non‐uniform borders of brain tumors.

#### 2.1.4 Support Vector Machine

In order to address challenges in supervised classification [67], a kernel‐based parametric approach called SVM was implemented. This method proved to be exceptionally valuable when dealing with multi‐domain classification tasks and offers a dependable approach to binary classification. Despite its immense classification capabilities, applying SVM in practical scenarios can be demanding both in terms of computation and understanding. SVM has been widely utilized in the segmentation of brain tumors because of its outstanding classification ability. It affords the advantage of facilitating the detection and localization of brain tumors within the MRI image, further enabling the segmentation of tumor cells to compute the size of the tumor area within the segmented region [68].

Kharrat et al. [69] also carried out another research study on brain tumors, in which they developed an approach by incorporating a genetic algorithm (GA) to support vector machine (SVM) in searching for the best characteristic of brain tissue through MRI data employing the spatial gray level. The GA selected these most pertinent traits and then provided them to the SVM for the classification of tissues into normal, malignant, or benign tumors.

#### 2.1.5 Clustering‐Based Segmentation

**FIGURE 9:**
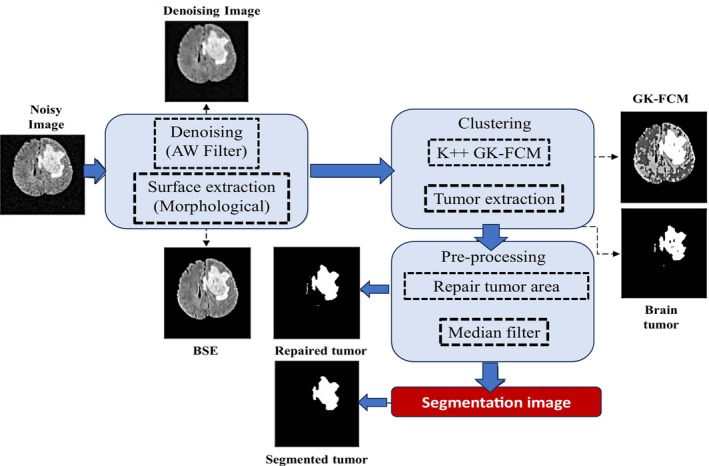
The clustering‐based brain tumor segmentation technique. It contains preprocessing and post‐processing modules. It uses the GKFCM clustering technique to find the tumor area [70].

The use of clustering for segmentation is an unsupervised technique in which it groups pixels/voxels with similar intensities while considering some commonly shared properties, as illustrated in Figure 9. This technique partitions different pixels into well‐defined groups [27, 71]. Generally, two types of clustering strategies are used, that is, hard clustering and soft clustering. Hard clustering assigns one pixel/voxel to only one cluster according to the relatively common properties or intensities. On the other hand, soft clustering allows estimating the probability of a pixel/voxel's membership for two or more defined clusters.

- **Density‐based clustering:** The region's density of displayed pixels/voxels is utilized for density‐based grouping. Clusters arise in regions with a high density of comparable pixels, while low‐density pixels create continuous patches that separate these dense clusters. These patches, known as noise or outliers, are classified in various density‐based clustering approaches. The HDBSCAN algorithm, introduced by Campello et al. in 2013, is a hierarchical density‐based clustering approach that constructs a comprehensive cluster hierarchy. This hierarchy can subsequently be condensed to include only the most significant clusters.
- **k‐Means clustering:** The k‐means clustering algorithm divides the input data into k groups by measuring the distance between data points; it is one of the essential features in pixel‐based methods [27, 72]. In this, high similarity exists in clusters and low similarity between clusters. Finally, to segment the image, appropriate clusters are assigned to each pixel/voxel.
  Alam et al. [73] had come up with a new technique for detecting brain tumors in MRI images. They proposed that template‐based k‐means should combine with an augmented FCM (fuzzy C‐mean) to form an algorithm. Segmentation via this template‐based k‐means will choose the best template according to the gray‐level intensity of the image. The study results indicate that the technique differentiates healthy and diseased brain tissues without modifying gray‐level intensity. Additionally, the method is faster than other technologies in detecting brain malignancies. In 2015, Liu and Guo [74] also designed a similar clustering technique that segmented brain MRI images.
- **FCM clustering:** The most essential and well‐known approach in MRI is the FCM clustering method, which has been widely employed [75]. It involves categorizing pixels in an image into multiple classes and assigning membership to each pixel unit based on its proximity to the class centers. Pixels can now belong to more than one class. The FCM technique is very suitable for data with numerous cluster solutions due to the use of fuzzy membership functions [34, 76].

#### 2.1.6 K‐Nearest Neighbor (K‐NN)

The k‐NN approach is an essential and potent tool for data classification and segmentation, effectively classifies isolated pixels (or voxels in 3D images) by comparing them to their neighboring pixels. This is done by identifying the nearest unlabeled neighbor and utilizing their attributes to determine the correct class or label. This prediction method calculates the similarity or the distance between the training examples and the new test points that are unlabeled to predict the label of test points. This approach is pertinent and does not impart any runtime performance issues given current processing capabilities for systems [77].

#### 2.1.7 Random Forest

Random Forest (RF) is one of the practical techniques that solve segmentation issues associated with brain tumors. In particular, it effectively solves those issues related to high‐dimensional feature vectors, multi‐class classification, and HGG. Based on discriminated tumor spots, Koley et al. [78] classified patterns using RF. They used 86 different characteristics to quantify the gathered lesions, which were then used as input for the classifier in the formation of the training dataset. Another innovative RF‐based approach was proposed by Goetz et al. This approach employed a domain adaptation technique to overcome sample selection errors caused by sparse sampling. By making use of sparse and unambiguous annotations, this suggested strategy improves the development of high‐quality classifiers for a variety of tissue classifications.

#### 2.1.8 Markov Random Field (MRF)

**FIGURE 10:**
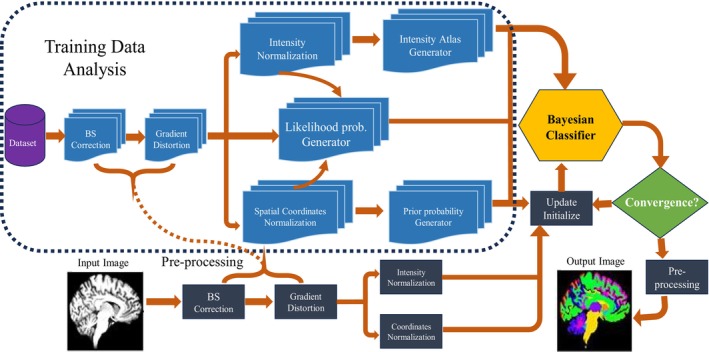
Multilateral Markov technique for brain segmentation. It uses a pre‐processing module that has gradient distribution and bias field correction. The Bayesian classifier is used to segment the different areas in the brain.

Besagt introduced this method in 1975 [79] and later modified it by Geman in 1984 for applications with images. Since its introduction, several algorithms based on MRF have been proposed for image segmentation, mainly in MRI brain segmentation [80]. Ahmadvand and Yousefi [81] have developed algorithms to consider multiple aspects of the image, including context, intensity, texture, spectral properties, and geographical information in clustering. The MRF approach is regarded as efficient because it reduces the probability of overlapped noise that is influential on the result of clustering, as shown in Figure 10.

#### 2.1.9 Active Contour Models

The segmentation approaches mentioned earlier have achieved significant success in extracting brain tumor borders from 2D MRI data. In 3D image segmentation, model‐based techniques are frequently employed. These strategies involve using geometric deformable models and parametric deformable models. These models use previously available information on the shape, position, and orientation of objects to form a connected and continuous model for a given anatomical structure [82]. The extraction of structures from medical imaging and the reconstruction of their geometric representation is not so easy, as is the case with tumor formation due to the complex and diverse anatomic model observed. A 2D or 3D curve or surface is called a deformable model, which is deformed using a speed function influenced by local and global forces. The choice of model attributes that are local or global depends on whether one aims to identify object boundaries or other features in an MRI image.

- **Parametric active contour models:** Active contour models, often called snakes, are widely used in brain tumor segmentation applications due to their effective handling of inherently unpredictable biological structures. Furthermore, they allow for the segmentation and tracking of anatomical structures in MRI images by exploring the impossibility of the image data together with the prior knowledge about the size, position, and shape of the structures [25].
- **Geometric deformable models:** Thereby, the mechanisms of breaking and joining contours are highly complex for researchers to resolve in volumetric 3D image segmentation. Researchers have designed geometric deformable models based on curve evolution theory and level sets. According to Malladi et al., these models have a solution to this problem [83, 84]. However, while applying these models in segmenting tumors from MRI images, the result in terms of consistency of object shape becomes challenging with the standard issue faced by many segmentation algorithms [83, 85, 86].

### 2.2 Deep Learning Techniques

Deep learning techniques are state‐of‐the‐art in medical image segmentation. This is due to the advancements in deep learning algorithms, a large amount of datasets, and powerful GPU‐enabled computing devices [87, 88, 89, 90]. The minimum computational requirements needed to run widely used architectures such as U‐Net, ResUNet, and transformer‐based models require modern GPUs with at least 8–16 GB RAM (e.g., NVIDIA RTX 3080 or A100), RAM capacities, and potential reliance on cloud platforms (like Google Colab or AWS for large foundation models). Researchers have been particularly captivated by the potential of CNNs for accurately segmenting brain tumors and detecting tumor sites. To better understand CNN‐based approaches for brain tumor segmentation, the authors have presented a comprehensive list and description of these approaches in Table 3. The aim of this section is to emphasize the architectural elements of these models and highlight the distinctive characteristics incorporated in each design.

**TABLE 3:** Overview of deep learning‐based brain tumor segmentation methods.

Ref	Year	Algorithm	Segmentation
[91]	2025	GAN CNN	MRI brain tumor
[87]	2024	UNet and DCNN	MRI brain tumor
[92]	2023	3D AGSE‐VNet	MRI brain
[93]	2023	Advances CNN	Medical image
[87]	2022	Joint CNN	MRI brain
[94]	2022	Hybrid CNN	MRI brain
[95]	2022	Two‐stage cascaded	Brain tumor
[96]	2021	Multi‐path 3D CNN	MRI brain tumor
[97]	2021	DIU‐Net	MRI brain tumor
[98]	2020	DCU‐Net	Brain tumor
[99]	2019	Sub‐region CNN	Brain tumor
[100]	2019	FCN	MRI brain
[101]	2017	Cascaded CNN	MRI brain
[102]	2017	Cascaded CNN	MRI brain
[103]	2015	FCN CNN	Medical image

#### 2.2.1 Based on Fully Convolutional Network Approach

**FIGURE 11:**
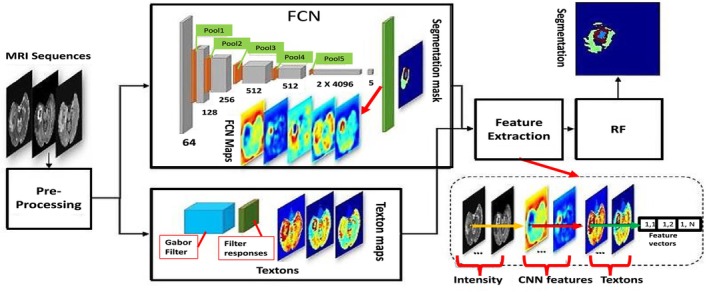
A fully convolutional network (FCN) technique for brain tumor segmentation, which has three parts: (1) pre‐processing, (2) convolutional network, and (3) high‐level feature extraction [105].

Long et al. [104] introduced the concept of a fully convolutional network (FCN). In their study, they suggested replacing the classifier layer of regular convolutional neural networks (CNNs) with dense FCN layers [103]. This substitution involved replacing fully connected layers with 11 convolutional layers [105]. However, the FCN approach only utilized local information, resulting in a loss of global semantic context in the images and therefore leading to ambiguous segmentation, as shown in Figure 11. To address this issue, the researchers combined the VGG‐16 basic model with the FCN model. This integration proved helpful in achieving more effective segmentation results by eliminating connections that could compromise the fusion of low‐layer and high‐layer characteristics in the final layer. This combination of models improved the segmentation process.

Sun et al. [96] proposed a 3D architecture with multiple pathways for segmenting gliomas in MRI images. Each pathway employed an FCN and integrated 3D dilated convolutions. This method simplifies the extraction of various receptive fields from multi‐modal MRI images. The resulting feature maps are spatially integrated using skip connections. This approach holds promise in improving the accuracy of FCN models in detecting tumor region boundaries.

#### 2.2.2 Cascaded CNN Approach

**FIGURE 12:**
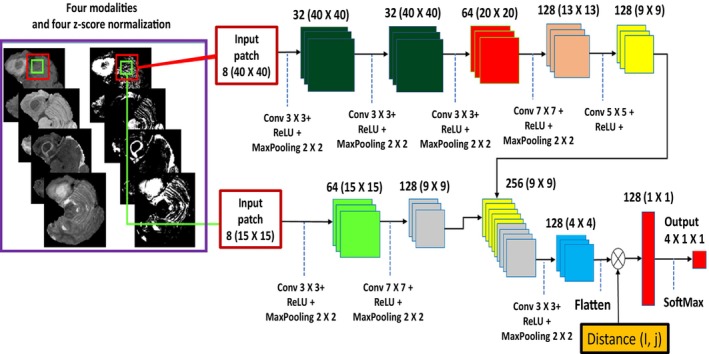
A cascade network for brain tumor segmentation. The local and global patches are represented by the green and red windows inside the input MRI images, respectively. The dynamic window approach (DWA) module is shown near the structure's conclusion, before the FC layer [95].

**FIGURE 13:**
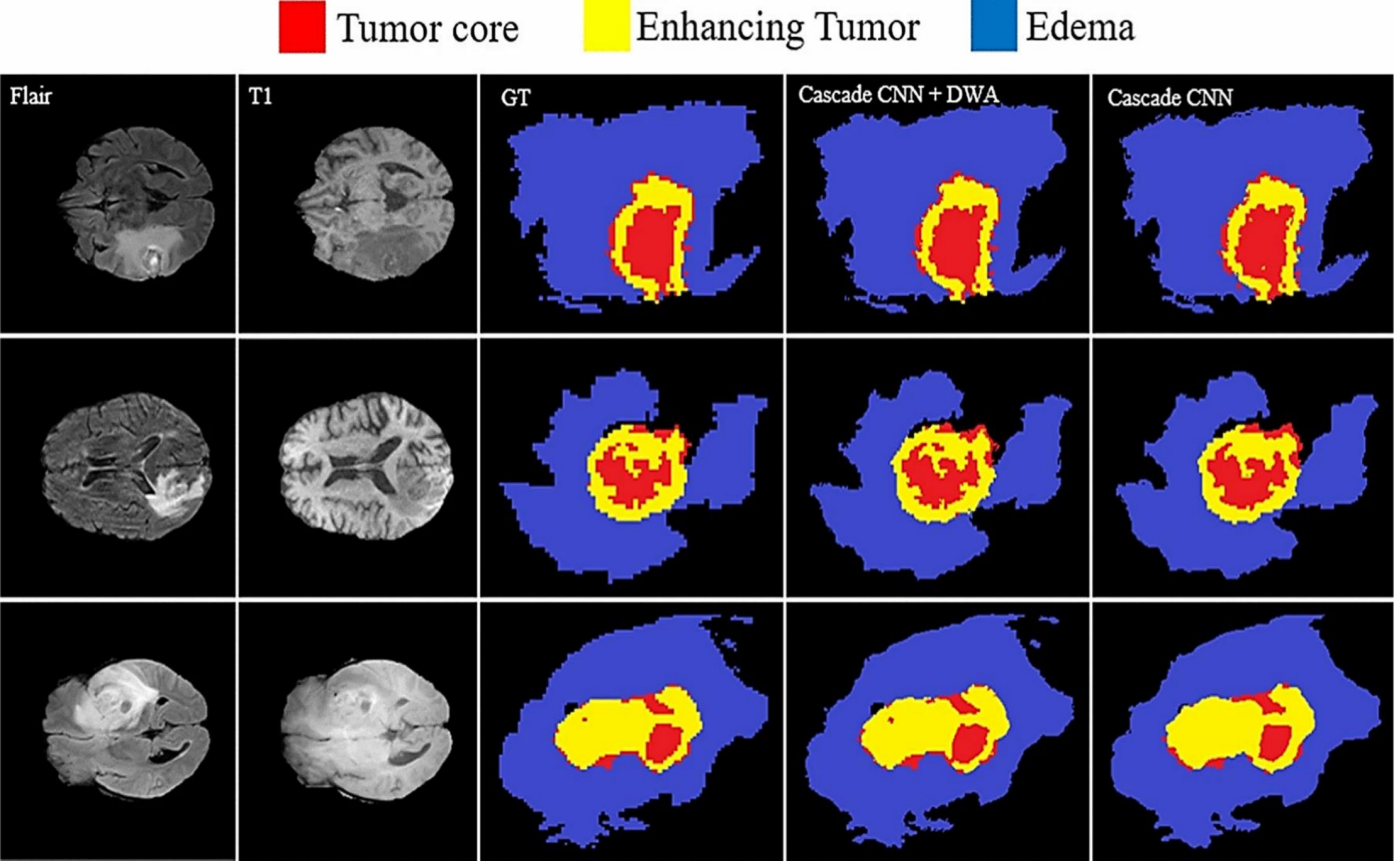
The brain tumor segmentation results using cascade‐based CNN structure, where columns 1 and 2 are inputs, and column 3 is ground truth. Columns 4 and 5 show edema, enhanced, and core areas, which are represented by the blue, yellow, and red, respectively [95].

More computationally intensive Clinical joint segmentation models [87] usually use the cascaded architecture. This is where a series of connected CNNs are constructed and the inputs to different layers of other CNNs are outputs from a CNN, see Figures 12 and 13. This methodology has shown its potential in improving systems that generate independent multiscale predictions, such as dual‐path algorithms. These methods embed contextual information in other CNNs as additional image channels by utilizing an input cascade [101, 102].

The cascade architecture concatenates local routes and mixes the output of the first CNNs with the first hidden layer of the second CNN [101]. Another type of cascade design, hierarchical subregion segmentation, divides the multiple‐class segmentation problem into a series of binary segmentation problems [99]. Then, the segmentation of brain tumor subregions is done progressively. In this approach, the hierarchical structure of brain tumor subregions is utilized to reduce false positives and associated concerns about class imbalance.

In Wang's work [99], three hierarchical networks were employed to separate subregions of brain tumors. First, it isolated the whole tumor, created a bounding box using multimodal MRI data, and passed the bounding box to the second network that proceeded to segment the core of the cancer. A bounding box for the output of TNet was made on the tumor's core, which was then input into the third network called ENet. It handled the segmentation of the enhancing part of the tumor. Wang et al. [99] extended this further by putting test‐time augmentation to improve the ability of segmentation results in uncertainty estimation and adding more postprocessing using CRF. Further tests were also conducted for validation of the efficiency of the pipeline to be segmented.

#### 2.2.3 Based on Dilated Convolution Approach

A modified method of CNNs, known as dilated convolution or atrous convolution, was created by Yu and Koltun to improve segmentation [106]. This technique effectively compiles multi‐scale contextual data without sacrificing resolution. In contrast to conventional pyramidal CNNs, the dilated approach enables linear parameter increase and exponential expansion of receptive fields while still preserving spatial information. As a result, it yields more effective input data features compared to regular convolution with the same parameters. The utilization of a dilated‐based model offers the advantage of retaining the spatial resolution of the image, thereby facilitating a dense forecast. However, the drawback is that dilated convolutions tend to separate image pixels from their surrounding environment, making them more prone to diagnostic errors.

Chen et al. proposed an improved and efficient 3D DMFNet for real‐time dense volumetric segmentation. This network was constructed using dilated multi‐fiber (DMF) and multi‐fiber (MF) units. In their previous work from 2018, the authors developed a lightweight 3D convolutional network based on the MF unit, which has now been augmented with the inclusion of the 3D DMF unit. This enhancement resulted in reduced computational costs without compromising the accuracy of brain tumor segmentation. The channel grouping technique is a crucial component of the DMF unit, as it aims to decrease the connections between feature maps and kernels by grouping the convolutional channels.

The DIU‐Net architecture [97] includes a multiplexer to support inter‐fiber communication. Additionally, dilated fibers were used to scale up the contextually relevant field and to drag multi‐scale 3D spatial correlations of lesions of brain tumors. The inception modules with dilated convolutions in both the contracting and expanding routes are adopted within the design of the proposed DIU‐Net architecture, following the U‐Net design. This enables the extraction of both local structural information and global contextual information. After these three 11 convolutions of the process in the dilated inception modules, there is a l‐dilated convolutional filter with l taking on values of either 1, 2, or 3.

#### 2.2.4 Based on the Top‐Down/Bottom‐Up Approach

The structure of this model consists of the encoder and decoder stages, also known as the downsampling and upsampling stages, respectively. The main objective of this design is to create two segmentation channels. The initial process involves extracting features from images and reducing their dimensionality using a sequence of convolutional and pooling layers. Alternatively, convolutional layers with increasing step lengths can be used instead of the pooling layer to achieve the same reduction effect. This procedure results in low‐resolution feature maps.

On the contrary, the second route is run in a counterclockwise direction compared to the first route. Deconvolution layers, also known as fractionally stridden convolutional or convolutional transposition layers, are employed to increase the dimensions of the image. This aids in accurately identifying the desired class. In specific cases, these pathways are placed between unpooling layers or intermediate feature maps.

The U‐Net design and the V‐Net architecture [107] are two well‐known models for general medical segmentation. The U‐Net design was further used for brain tumor segmentation, while Zhang et al. [93] proposed the ME‐Net architecture to enhance the V‐Net design. The ME‐Net architecture consists of four encoder structures for segmenting MRI modality images. It improves upon the V‐Net design, which only has one down‐sampling path. The feature maps created from these images are then transmitted to the decoder structure through skipping connections. To compensate for any loss, the feature maps from the appropriate downsampling step are also included in the upsampling process.

Baid et al. [100] proposed a U‐Net‐based automatic segmentation design for brain tumors. The designed approach compensates for class imbalance in the tumors by subtracting a weighted patch from the tumor boundaries. This way, detected subregions of the cancer are classified into separately segmented regions. Additionally, the performance of the segmentation technique that relies on weighted patches is competitive with the performance of the pixel‐based approach, particularly in cases of narrow borders between tumor subparts.

By integrating 3D CNNs into a U‐Net architecture [108], Ali et al. successfully developed a segmentation model that surpassed previous methods. This advancement led to improved segmentation results and more accurate forecasts. The initial framework utilized 3D CNNs, originally introduced by Chen et al. [109]. To capture multiscale features for volumetric segmentation, the model employed MF blocks and adaptive weighted dilated convolutions. Additionally, it effectively preserved and transmitted spatial information. The hyperparameters of the second design, named the 3D U‐Net, were modified from the traditional U‐Net. This design outperformed various networks in the BraTS 2018 competition.

To address these challenges posed by intra‐tumoral architectural heterogeneity, Pereira et al. [110] presented a deep CNN‐based automatic segmentation model. This model has a more complex design that integrates specific instances for the segmentation of high‐grade glioma and low‐grade glioma. However, the authors found that increasing the depth alone did not improve results for LGG cases. The recommended architecture for LGG cases was much simpler, with only 4 layers. This included one fully connected layer to the input and two others fully connected as output. In contrast, the recommendation for HGG cases included far more layers. In total, there were 11 layers with three fully connected layers, two max‐pooling layers of 3×3 filter size and 2×2 stride, and six convolutional layers of a 3×3 filter size and 1×1 stride. On the other hand, the LGG‐CNNs used a total of nine layers: three fully connected, two max‐pooling, and four convolutional layers with filter sizes of 3×3 and 1×1, respectively. Rehman et al. [111] introduced an approach in the form of BU‐net based on the encoder‐decoder framework to further investigate brain tumor segmentation. This BU‐net model is distinguished by its incorporation of residual prolonged skip and broad context in the U‐Net.

The architecture of BrainSeg‐Net [112] has four blocks and one single transition block that includes an encoder route and a decoder route. There are connected encoder and decoder blocks that interconnect with the FE block. It concatenates the output of all the deeper‐level FE blocks with the output of the transformation layer, which is relevant to the FE block in the decoder block. This would help to improve the segmentation capabilities of the FE block and, at the same time, facilitate the detection of small tumor regions. In addition, it also recovers small features that are lost during downsampling through the skip connection. The architecture of BrainSeg‐Net was good enough to record good segmentation results on BraTS2017, BraTS2018, and BraTS2019 benchmark datasets.

The DeepMedic architecture, proposed by Kamnitsas et al. [113], uses 11‐layer 3D CNNs with residual connections. It is composed of two parallel convolutional circuits to handle the input effectively at different sizes. This leads to less computation and ensures that a good deal of receptive field is adequately covered for correct categorization. Casamitjana et al. proposed two architectures of fully convolutional 3D CNNs, and Kamnitsas et al. [113] trained one model that impersonates their three‐pathway DeepMedic net. The first is called $3{\text{DNet}}_{1}$, based on a VGG‐inspired 3D FCN network using skip connections to attach high and low‐layer information. The second is called $3{\text{DNet}}_{2}$, which relies on a 3D version of the network proposed by Ronneberger et al. The $3{\text{DNet}}_{3}$ network is the modified version of the DeepMedic network [113].

The two‐path architecture allows the collection of high and low‐resolution data from the input segment. Different input sizes are used in each pathway. Besides, the second model uses Adam optimization, batch normalization, and the ReLU activation function. Deep learning has become a popular method for segmenting brain tumors in MRI images, largely due to the effectiveness of convolutional neural networks. CNNs are considered an efficient means of extracting data and learning representations from images, as highlighted by Sultana et al. [105]. The architecture of CNNs can vary depending on the segmentation technique employed, such as single, instance, or semantic segmentation.

### 2.3 Hybrid Techniques

**FIGURE 14:**
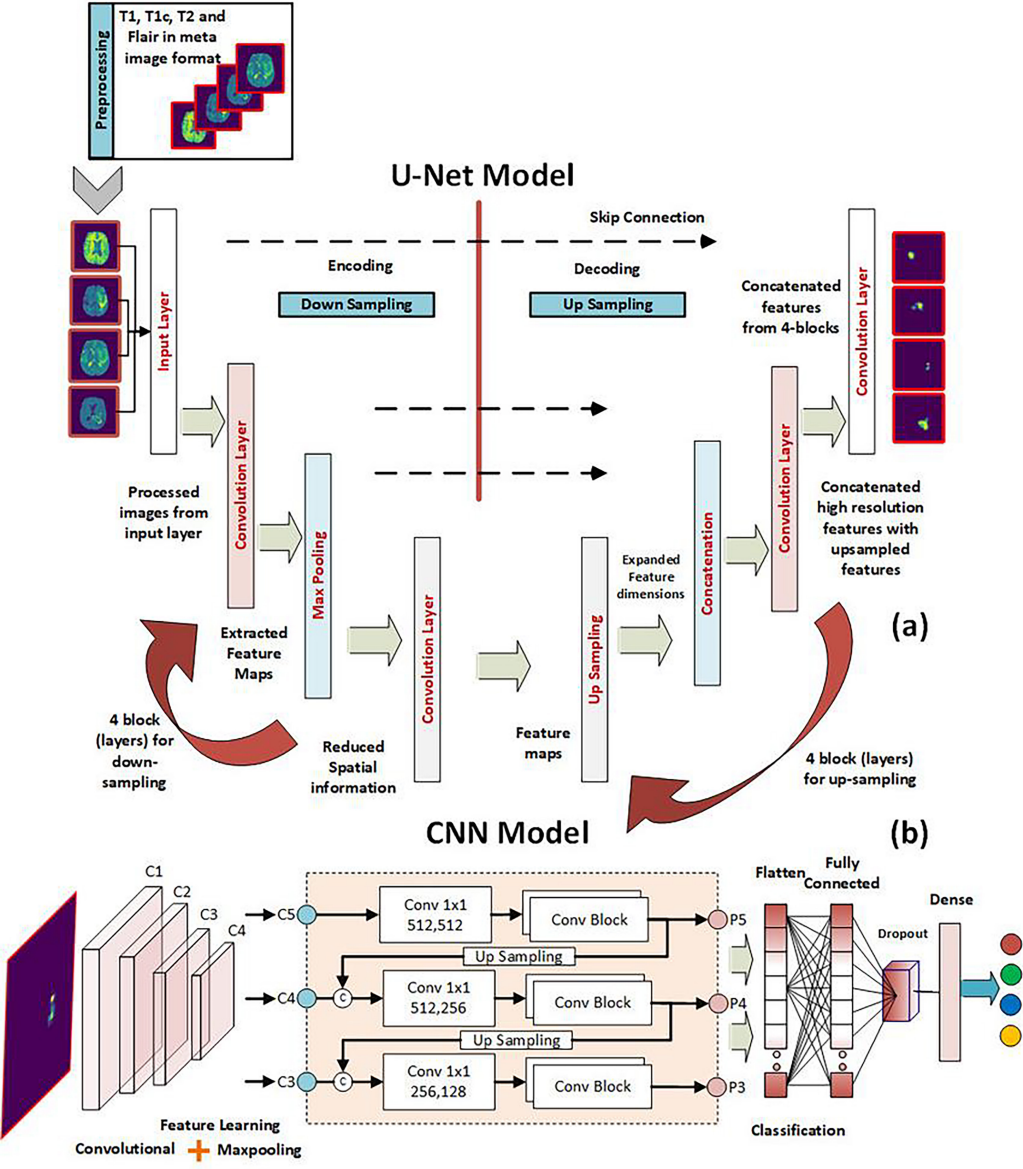
The combination of a U‐Net and CNN architecture for segmenting and classifying brain tumor MRI. (a) U‐Net layers for brain image segmentation. (b) The CNN layers are used for the segmented ROI in MRI [87].

**FIGURE 15:**
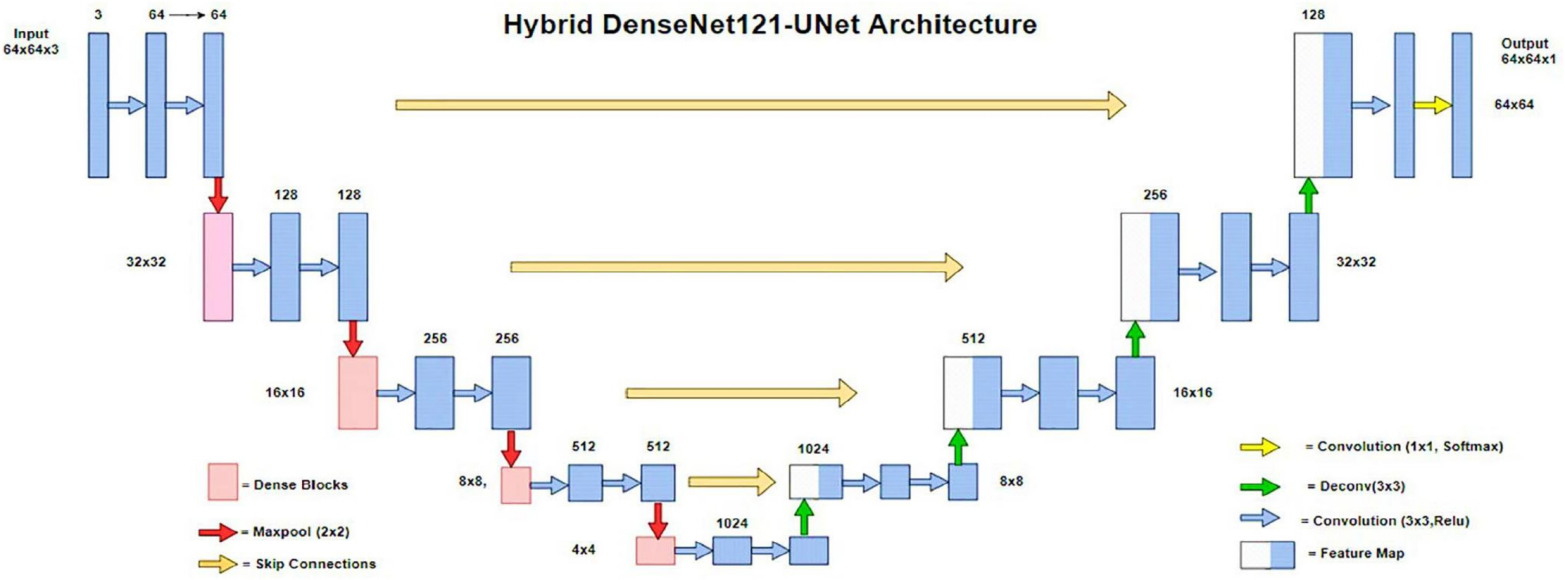
The Hybrid DenseNet21‐Unet approach for brain tumor segmentation. It is divided into two parts: In the first part, the encoder utilizes the DenseNet121 model, while in the second part, the decoder employs the UNet model [114].

Hybrid techniques are needed because they can use two or more methods and maximize the benefits of segmenting medical images, which could result in reliable outcomes at the end of the procedure (Figure 14). The fusion of multiple techniques has shown outstanding promise in improving accuracy significantly [94, 114, 115], as shown in Figure 15. Table 4 summarizes recent hybrid approaches in brain tumor segmentation in MRI images.

**TABLE 4:** Overview of hybrid learning‐based brain tumor segmentation methods.

Ref	Year	Algorithm	Segmentation
[116]	2023	FCM and K‐mean	Brain tumor
[117]	2023	Advances in TML	Brain tumor
[118]	2022	FCM and K‐mean	Brain tumor
[119]	2022	FCM and K‐mean	Brain tumor
[118]	2018	FCM and K‐mean	Brain tumor
[119]	2021	Support vector machine	Brain tumor
[118]	2020	FCM and K‐mean	Brain tumor
[119]	2019	FCM and K‐mean	Brain tumor
[120]	2019	FCM & CNN	Brain tumor
[121]	2019	FCM	Brain tumor
[71]	2019	K‐mean and FCM	Brain tumor
[114]	2022	DenseNet	Brain tumor

Parveen and Singh had previously carried out work that showed a hybrid model of SVM with FCM to effectively classify the tumor region in brain images [122]. The methods used for enhancement for better contrast of the images have been applied before removing the skull using the techniques of dual‐threshold and morphological. FCM clustering was then applied to segment images for detecting suspicious spots in brain MRI data. The brain MRI images were classified through the SVM method after the features were extracted using the gray‐level run‐length matrix technique. Consequently, the categorization of brain MRI images yielded more accurate and effective results.

Akselrod‐Ballin et al. developed a hybrid method that combines multi‐scale segmentation weighted aggregation with an SVM‐based classification technique to detect the brain structures in MRI. This method utilizes an amorphous pyramid to display the hierarchical structure created through segmentation. The pyramid's flexible and adaptive presentation allows for the recognition of various anatomical elements of different sizes. In a study by Nyma et al. [123], a hybrid Otsu thresholding and FCM image segmentation method was proposed. Before using FCM to divide the brain MR images into distinct regions, Otsu thresholding was employed as a coarse segmentation approach to identify homogeneous regions in the input image.

Vaibhavi and Rupal proposed a hybrid technique that combined the k‐means and FCM algorithms in order to segment brain tumors in MRI images in 2018. The accuracy of their approach was compared to the independent findings of the k‐means algorithm and FCM algorithm. To detect brain tumors in MRI images, Hamad et al. [121] proposed a hybrid technique that integrates the FCM algorithm with a threshold method. This hybrid approach addresses the need for noise reduction and enhances image contrast. The procedure incorporates a balanced contrast enhancement mechanism. In MRI scans, the FCM methodology is used to classify the normal part of the brain, while the threshold method is employed to transfer the improved image into a binary format for tumor region segmentation. As a further step, tiny edges are detected using the Canny edge detection approach.

Zhang and colleagues suggested a hybrid clustering technique for segmenting brain tumors in their study [124]. They combined morphological procedures with the k‐means++ and FCM algorithms to achieve this. Non‐brain tissue was eliminated from an MR brain image using these methods to reduce noise. They employed the Gaussian kernel‐based FCM algorithm for clustering, which improved the accuracy of tumor classification. To avoid overfitting, they utilized k‐means++ to establish cluster centroids.

### 2.4 Evaluation Metrics

Most assessment metrics for image segmentation are created using a confusion matrix, also known as a contingency table [125]. This table shows the total number of predicted pixels and the total number of ground truth pixels for each class. Table 5 provides a detailed overview of the structure of the confusion matrix, with $G_{y}P_{y}$ representing the ground truth value for the pixels and $P_{y}$ representing the predicted value.

**TABLE 5:** Confusion matrix to evaluate the segmentation results with respect to the ground truth values.

Ground truth/prediction	1	2	3
1	G_1_ *P*_1_	G_1_ *P*_2_	G_1_ *P*_3_
2	G_2_ *P*_1_	G_2_ *P*_2_	G_2_ *P*_3_
3	G_3_ *P*_1_	G_3_ *P*_2_	G_3_ *P*_3_

The confusion matrix shows the number of pixels for each situation. For example, the cell $G_{1}P_{2}$ indicates the number of pixels that were predicted as class 1 but actually belong to class 2. True positive (TP) predictions are found when the row and column indices match, meaning they are on the diagonal of the matrix. Cells in the image that are missing represent false negative (FN) predictions, where the model predicted a different class than the actual ground truth class. Conversely, the columns show the predicted labels for each class, and any deviation from the diagonal within a column indicates a false positive (FP) prediction.

#### 2.4.1 Dice Coefficient

To evaluate how well algorithms segment organs or other structures in MRI, CT, and OCT images, the Dice coefficient is often used in medical image analysis [2]. The Dice coefficient measures the similarity between two sets of data by determining how much they overlap. It is usually shown as a fraction between 0 and 1, with 1 indicating a perfect match.

#### 2.4.2 Hausdorff Distance

The use of Hausdorff distance is increasingly becoming popular as a metric for medical image segmentation [126]. Unlike statistical metrics, the Hausdorff distance focuses on the boundary pixels within each patch of pixels that belong to the same class. It measures the distance between corresponding points on different borders, regardless of whether there is a single border or multiple borders present. However, the Hausdorff distance only considers the maximum distance between two points, according to its definition. In contrast, the average Hausdorff distance takes into account all the border pixels within a patch.

(3) $$\mathrm{HD}\left(\mathrm{Gt},\mathrm{Pd}\right)={\text{mean}}_{p_{p}d\in \mathrm{Pd}}\min_{p_{g}t\in \mathrm{Gt}}{\left\|p_{\mathrm{gt}}-p_{\mathrm{pd}}\right\|}^{2}$$

where $p_{\mathrm{pd}}$ is predicted pixels and $p_{\mathrm{gt}}$ is ground truth pixels during segmentation.

## 3 Performance Comparison

**FIGURE 16:**
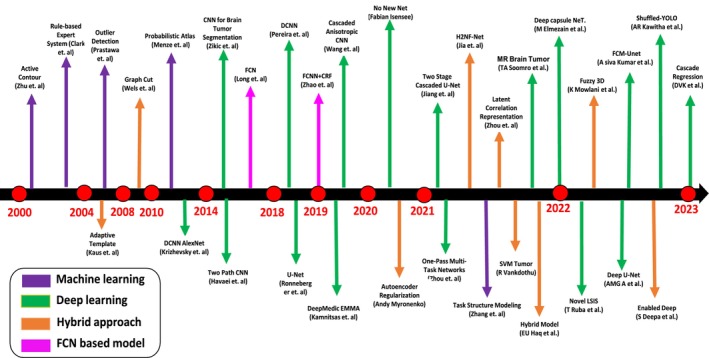
The four different brain segmentation techniques: machine learning, deep learning, hybrid, and FCN‐based approach. Each technique offers unique advantages and uses various algorithms to accurately segment brain structures. Researchers must carefully consider the strengths and limitations of each approach when deciding which technique best suits their specific requirements.

**FIGURE 17:**
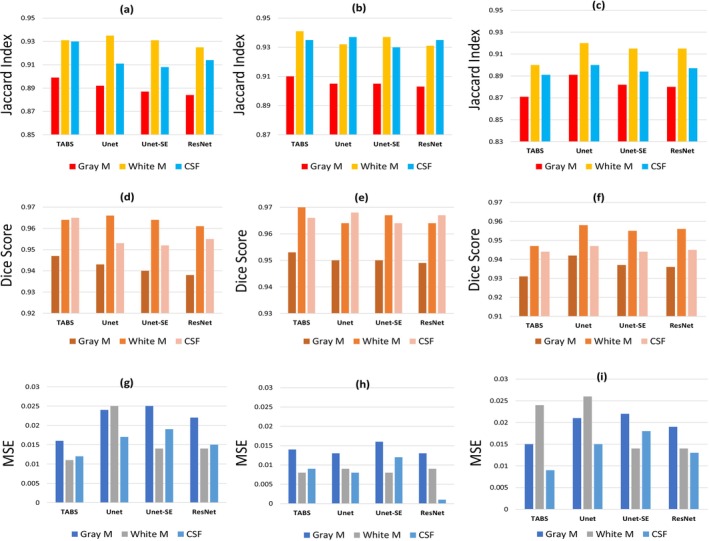
The Jaccard index, Dice score, and MSE values of different brain segmentation techniques [127]. The Jaccard index is represented by (a), (b), and (c) for datasets 1, 2, and 3. The Dice scores of distinct brain segmentation approaches on three separate datasets are shown by (d), (e), and (f). The (g), (h), and (i) values represent the mean square errors of three distinct approaches on three different datasets.

**FIGURE 18:**
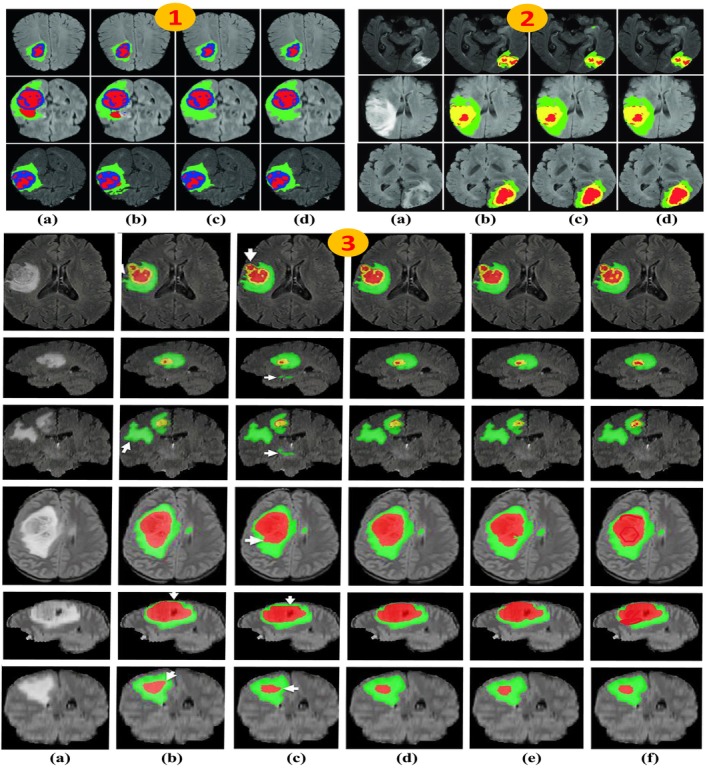
The qualitative analysis of brain tumor segmentation in MRI images. In panel (1), (a), (b), (c), and (d) represent the 3D‐Unet, Kao et al., DMF‐Net, and ground truth, respectively. In panel (2), (a), (b), (c), and (d) represent the ground truth, U‐net, Unet2, and ResU‐Net, respectively [128]. In panel (3), (a), (b), (c), (d), (e), and (f) represent the GT, Iso‐3D Net, A2.5D‐Net, 3D + MVF, U‐net + CNN, and Hybrid‐Net21, respectively [129].

In this section, we have discussed both the quantitative and qualitative analysis of advanced MRI image segmentation techniques for brain tumors. Figure 16 provides detailed information about the selected approaches. The quantitative comparison among different approaches is shown in Figure 17, and the qualitative comparison among the different approaches is shown in Figure 18. We cover commonly used assessment metrics, performance evaluations, and provide a comprehensive discussion on the subject.

## 4 Brain Tumor Data Sets

Several publicly accessible datasets [130] have made significant contributions to brain tumor segmentation research by offering consistent standards with high‐quality imaging data and expert annotations. Among the most popular is the Brain Tumor Segmentation (BraTS) collection, which provides multi‐institutional preoperative MRI images with annotated tumor subregions such as enhancing tumor, peritumoral edema, and necrotic core. BraTS has played a key role in annual competitions that push the boundaries of algorithm development and clinical translation. Another useful resource is The Cancer Imaging Archive (TCIA), a large‐scale, NIH‐supported archive that contains a variety of brain tumor imaging collections, including LGG‐GBM, TCGA‐GBM, and TCGA‐LGG datasets, which frequently supplement BraTS in research.

Along with these datasets, a variety of open‐source tools have arisen to aid annotation, visualization, and benchmarking efforts in the brain tumor segmentation procedure. ITK‐SNAP [131, 132] is a popular interactive software application for manually segmenting anatomical components in 3D medical images. It supports semi‐automated region expansion and active contour approaches. 3D Slicer [133] is a more comprehensive platform that includes a variety of functions such as visualization, registration, multi‐modal fusion, and segmentation, as well as extensibility via its extensive module ecosystem. For robust benchmarking and baseline model training, nnU‐Net (“no‐new‐Net”) has become the de facto standard, providing a self‐configuring deep learning pipeline that adjusts to dataset‐specific requirements without manual tuning.

## 5 Discussion

In this section, we analyzed the performance of machine learning, deep learning, and hybrid approaches in terms of dice coefficient, Haussdorff distance, Dice score, Jaccard index, and mean squared error (MSE) for comparison.

### 5.1 Machine Learning Techniques

Machine learning techniques have some drawbacks, such as needing pre‐feature extraction before modeling and lacking high‐level features during segmentation. Different applications of these techniques require pre‐processing methods to isolate the region of interest in MRI images. For example, in [118], they use initial features of the gray‐level co‐occurrence matrix and save them using the RFC technique. This shows that several challenging stages are necessary before training an algorithm. Another drawback is the processing time of these techniques. These algorithms often perform segmentation tasks more slowly compared to deep learning methods, as shown in Tables 6, 7, and 8.

**TABLE 6:** The performance comparison of multi‐network approaches for imbalance issue in data.

Multi networks driven approaches						
Methods	Dice			Hausdorff		
	WT	TC	ET	WT	TC	ET
[27]	0.9	0.84	0.78	3.89	6.48	3.28
[134]	0.9	0.84	0.77	5.18	6.28	3.51
[135]	0.86	0.73	0.72	7.5	9.5	5.7
[136]	0.91	0.85	0.79	4.18	4.97	26.57
[137]	0.88	0.79	0.72	29.21	11.06	7.93
[3]	0.9	0.81	0.78	4.32	6.28	3.7
[138]	0.91	0.86	0.8	4.26	5.43	3.14
[139]	0.89	0.79	0.75	6.39	14.07	36
[140]	0.9	0.8	0.74	4.23	6.56	4.5
[141]	0.9	0.84	0.76	—	—	—
[142]	0.89	0.79	0.75	—	—	—
[143]	0.89	0.77	0.77	4.4	15.3	29.4
[144]	0.85	0.7	0.65	25.24	21.45	17.98
[145]	0.91	0.81	0.76	4.34	9.39	27.16

**TABLE 7:** The performance comparison of multi‐task approaches for the imbalance issue in data.

Multi tasks driven approaches						
Methods	Dice			Hausdorff		
	WT	TC	ET	WT	TC	ET
[146]	0.91	0.86	0.81	4.17	6.54	2.71
[147]	0.85	0.68	0.58	—	—	—
[148]	0.88	0.71	0.73	—	—	—
[149]	0.89	0.72	0.73	—	—	—
[150]	0.91	0.87	0.82	4.52	6.85	3.92
[151]	0.85	0.78	0.75	7.98	8.25	5.76
[152]	0.92	0.88	0.88	12.4	16.09	8.71

**TABLE 8:** The performance comparison of customized loss‐function approaches for the imbalance issue in data.

Customized loss function driven approaches						
Methods	Dice			Hausdorff		
	WT	TC	ET	WT	TC	ET
[153]	0.88	0.8	0.76	6.49	6.68	21.39
[3]	0.9	0.84	0.78	5.68	9.57	24.02
[154]	0.9	0.8	0.73	7	9.48	4.55
[131]	0.9	0.75	0.71	4.16	8.65	6.98
[155]	0.9	0.82	0.78	5.41	7.26	5.282
[156]	0.92	0.88	0.87	4.23	5.77	8.18

However, machine learning methods have an advantage in segmentation applications because they do not need labeled data for training. For instance, K‐means clustering is used to segment the relevant area in the image by grouping pixels based on their intensity, followed by a segmentation task during the post‐processing phase. As shown in Table 1, machine learning models generally perform well across various datasets.

### 5.2 Deep Learning Techniques

They are divided into three basic types: (1) fully supervised, (2) semi‐supervised, and (3) weakly supervised.

#### 5.2.1 Fully Supervised Deep Learning Techniques

The effectiveness of these techniques in a variety of tasks within MRI segmentation applications has been demonstrated in Figure 11. However, training deep learning networks for such tasks poses the challenge of class imbalance. In certain MRI applications where the region of interest, such as lesion regions in MRI images, is relatively small, deep learning algorithms find it difficult to accurately segment this restricted area.

To address this issue, a focal‐loss function approach is utilized in this model. The study on tiny area segmentation in MRI images emphasizes the significance of selecting the appropriate backbone network. Various backbones, such as modified Xception, ResNet101, and Inception‐V3, produce diverse outcomes, as evidenced in [4]. However, fully supervised approaches suffer from lengthy training times as a drawback. To enhance their performance on many photos, they require extensive training periods. For example, Lee et al. [157] mention that training a network usually takes about 7 days. Figure 18 shows that fully supervised techniques tend to have very low latency and achieve high scores in accuracy, recall, and F‐measure for MRI image segmentation.

#### 5.2.2 Semi‐Supervised Deep Learning Techniques

Semi‐supervised methods in MRI image segmentation have higher accuracy than machine learning algorithms (Table 6). In the RETOUCH dataset, a mean dice coefficient score of 76% was achieved with the semi‐supervised approach, compared to 73% without it [158]. Similarly, the semi‐supervised network achieved a mean dice coefficient score of 82% compared to 80% without it using a custom dataset, and a mean dice coefficient score of 93.04% compared to 92.85% without it in another study [159]. However, these methods struggle with segmenting small multi‐regions and are challenging to generalize across different datasets. Tables 6, 7, and 8 represent the quantitative comparison of multi‐network, multi‐task function, and customized loss function for supervised, semi‐supervised, and weakly supervised approaches, respectively.

#### 5.2.3 Weakly‐Supervised Deep Learning Techniques

These models are designed to segment MRI images without needing pixel‐level annotation. They are both efficient and effective for a range of applications. However, their complex structure and the requirement for pre‐processing and post‐processing steps present certain challenges. For instance, in [160], a weakly supervised segmentation model is proposed, which involves three training steps: classification, saliency map generation, and the level set technique. These models are limited to specific tasks and cannot be easily used for multiple tasks. Similarly, in [161], the technique relies on similar intensities between fluid and background regions, which may not be common in all MRI images. Figure 17 illustrates the qualitative results of brain tumor segmentation using different techniques. It shows that the segmentation results of deep learning and hybrid approaches are effective and reliable in brain tumor segmentation. These techniques efficiently segment the tumor lesion from the non‐tumor area in the brain MRI image.

## 6 Future Direction

Recently, there have been advancements in creating foundation models using large amounts of data and parameters. Chat‐GPT is a primary example, showcasing the potential benefits these models can have in our daily lives. Particularly in healthcare informatics, large AI models are revolutionizing approaches and utilizing the abundance of data available in the biomedical and healthcare sectors. This is crucial for creating and developing AI models for health‐related matters.

In clinical applications, it is crucial to explain the deep learning models to extract the high‐level features. To derive high‐level characteristics from different medical modalities, researchers should focus on machine learning and deep learning models. Established segmentation algorithms, such as the level set, effectively combine both machine learning and deep learning techniques to achieve strong results. Future research could explore integrating deep learning techniques with traditional segmentation methods.

Looking ahead, we might incorporate specific loss functions, like focused loss, into networks to tackle class imbalance issues. Existing methods can be adapted for various tasks. Reinforcement learning is an advanced technique for image segmentation. So, future studies can focus on this area to develop a universally applicable deep learning segmentation approach. To ensure the reliability of the models, techniques need to be evaluated on diverse datasets. Currently, techniques rely on private datasets, which calls for the availability of more publicly accessible datasets for segmentation. Investigating the credibility of brands adds an intriguing and beneficial perspective. Given the multitude and complexity of illnesses, specialists may annotate the same areas or pixels differently, making it difficult to distinguish what is right or wrong. By challenging the idealization of annotations, new ideas can be introduced to the field.

## 7 Conclusion

This paper provides an extensive overview of brain tumor segmentation techniques, focusing on traditional techniques, deep learning, and hybrid techniques. The advancements in medical imaging technology have generated large amounts of data that need accurate and efficient segmentation for diagnosis and treatment planning. Machine learning techniques like SVM (Support Vector Machines) and RF (Random Forest) have shown promise in segmenting brain tumors, but they struggle with capturing complex spatial relationships within the brain. Deep learning techniques, especially CNN (Convolutional Neural Networks) with unique architectures like U‐Net, V‐Net, and 3D CNNs, have revolutionized medical image segmentation by excelling at capturing intricate features and spatial information for accurate tumor segmentation. Hybrid approaches combining machine learning and deep learning have shown great potential for advancing the field and improving patient care. This survey highlights the advancements and challenges in brain tumor segmentation techniques, emphasizing the significant contributions of both deep learning and machine learning techniques to increase segmentation accuracy and efficiency. The choice of technique should be based on the specific requirements of the application and available data.

## Author Contributions

Khadija Bibi drafted the manuscript; Mehmood Nawaz supervised the study and polished the final draft; Sheheryar Khan, Muhammad Daud, and Anum Masood contributed to the analysis; Abdelgawad, Abbasi, Rizwan, and Ahsan Khan reviewed the draft, and Wu Yuan provided final suggestions and validated content.

## Conflicts of Interest

The authors declare no conflicts of interest.

## Data Availability

The data that support the findings of this study are available from the corresponding author upon reasonable request.
